# Host cellular unfolded protein response signaling regulates *Campylobacter jejuni* invasion

**DOI:** 10.1371/journal.pone.0205865

**Published:** 2018-10-15

**Authors:** Aya Tentaku, Takaaki Shimohata, Sho Hatayama, Junko Kido, Anh Quoc Nguyen, Yuna Kanda, Shiho Fukushima, Takashi Uebanso, Taketoshi Iwata, Kazuaki Mawatari, Nagakatsu Harada, Akira Takahashi

**Affiliations:** 1 Department of Preventive Environment and Nutrition, Institute of Biomedical Science, Tokushima University, Tokushima, Japan; 2 Bacterial and Parasitic Disease Research Division, National Institute of Animal Health, National Agriculture and Food Research Organization, Ibaraki, Japan; 3 Department of Nutrition and Metabolism, Institute of Biomedical Sciences, Tokushima University, Tokushima, Japan; 4 Department of Health and Nutrition, Faculty of Nursing and Nutrition, The University of Shimane, Shimane, Japan; University of Hong Kong, HONG KONG

## Abstract

*Campylobacter jejuni* is a major cause of bacterial foodborne illness in humans worldwide. Bacterial entry into a host eukaryotic cell involves the initial steps of adherence and invasion, which generally activate several cell-signaling pathways that induce the activation of innate defense systems, which leads to the release of proinflammatory cytokines and induction of apoptosis. Recent studies have reported that the unfolded protein response (UPR), a system to clear unfolded proteins from the endoplasmic reticulum (ER), also participates in the activation of cellular defense mechanisms in response to bacterial infection. However, no study has yet investigated the role of UPR in *C*. *jejuni* infection. Hence, the aim of this study was to deduce the role of UPR signaling via induction of ER stress in the process of *C*. *jejuni* infection. The results suggest that *C*. *jejuni* infection suppresses global protein translation. Also, 12 h of *C*. *jejuni* infection induced activation of the eIF2α pathway and expression of the transcription factor CHOP. Interestingly, bacterial invasion was facilitated by knockdown of UPR-associated signaling factors and treatment with the ER stress inducers, thapsigargin and tunicamycin, decreased the invasive ability of *C*. *jejuni*. An investigation into the mechanism of UPR-mediated inhibition of *C*. *jejuni* invasion showed that UPR signaling did not affect bacterial adhesion to or survival in the host cells. Further, *Salmonella* Enteritidis or FITC-dextran intake were not regulated by UPR signaling. These results indicated that the effect of UPR on intracellular intake was specifically found in *C*. *jejuni* infection. These findings are the first to describe the role of UPR in *C*. *jejuni* infection and revealed the participation of a new signaling pathway in *C*. *jejuni* invasion. UPR signaling is involved in defense against the early step of *C*. *jejuni* invasion and thus presents a potential therapeutic target for the treatment of *C*. *jejuni* infection.

## Introduction

*Campylobacter jejuni* is a Gram-negative microaerophilic bacterium that is a major cause of foodborne gastrointestinal illness in humans worldwide [1]. *C*. *jejuni* infection induces several intestinal inflammation-associated clinical symptoms, such as diarrhea, abdominal pain, and fever. Despite the severity of gastrointestinal symptoms, genomic studies have been unsuccessful in the identification of the specific virulence factors of *C*. *jejuni*. Nonetheless, previous *in vitro* studies revealed that multifactorial virulence factors participate in bacterial secretion, motility, adherence, and invasion in the pathogenesis of *C*. *jejuni*. In particular, the adhesion and invasion processes are closely associated with the ability of *C*. *jejuni* to induce inflammation, as defective adherence and invasion of *C*. *jejuni* strains were found to decrease the production of proinflammatory cytokines, such as interleukin (IL)-8, in cultured intestinal epithelial cells [2]. Hence, the adherence and invasion processes are key to the pathogenesis of *C*. *jejuni*.

Lipid rafts, which are enriched in cholesterol-specific microdomains within the plasma membrane of host cells, facilitate the invasion of *C*. *jejuni* and the molecular interactions between the host receptors and bacterial invasive factors, leading to activation of downstream signaling pathways in host epithelial cells. For this reason, treatment with methyl-β-cyclodextrin (MβCD), a compound that disrupts the formation of lipid rafts, significantly decreases the invasive abilities of *C*. *jejuni* [3]. Caveolar structures are thought to play a key role in lipid raft-mediated *C*. *jejuni* invasion, although some reports have suggested that several host cell-signaling molecules, such as phosphatidylinositol 3-kinase, protein kinase C, and mitogen-activated protein kinase, also take part in *C*. *jejuni* internalization [4–6], in addition to the Ca^2+^ and G protein signaling pathways [7].

During infection by *C*. *jejuni*, eukaryotic cell-signaling pathways are typically altered by the induction of several stress responses that are associated with the production of proinflammatory cytokines and caspase activation [8]. These host cell-signaling modifications are essential not only for initiation of symptoms, but also for the promotion of bacterial infection, especially adhesion and invasion. Thus, in *C*. *jejuni* infection, modulation of signal transduction in intestinal epithelial cells is expected to be a very strong candidate for treatment of *C*. *jejuni* infection. However, the activation of stress responses responsible for cellular signaling in *C*. *jejuni* infection remains poorly understood.

The endoplasmic reticulum (ER) plays a key role in several physiological functions, such as the synthesis, folding, and modification of most secretory and transmembrane proteins, lipid biosynthesis, and storage of intracellular Ca^2+^. Some environmental, pathological, and physiological stressors perturb ER homeostasis, resulting in the accumulation of both unfolded and misfolded proteins in the ER, as part of the ER stress response. Many studies have confirmed that ER stress is involved in the pathogenesis of a wide variety of diseases, including diabetes, cancer, neurodegenerative disorders, and inflammatory bowel disease [9–11]. To maintain ER homeostasis, the unfolded protein response (UPR) is induced in an attempt to decrease the accumulation of unfolded proteins by suppression of protein translation, normalization of protein folding, and promotion of ER-associated degradation [12, 13]. However, under conditions of severe ER stress, the cell is unable to maintain the protein homeostasis and UPR signaling switches to promote proinflammatory cytokines production and the induction of apoptosis [14].

In mammalian cells, UPR is well controlled by 3 transmembrane ER stress sensor proteins: protein kinase RNA-like ER kinase (PERK), inositol-requiring protein-1 (IRE1), and activating transcription factor-6 (ATF6). Under normal conditions, these sensor proteins bind to the ER-resident chaperone immunoglobulin binding protein (BiP), which is dissociated from them in response to ER stress [15–24]. This process leads to the activation of these sensor proteins and contributes to the transcriptional activation of UPR target genes to recover from ER stress. The ER is a critical stress-responsive organelle, where ER stress leads to production of nuclear C/EBP-homologous protein (CHOP), which has been implicated as a key mediator of ER stress-mediated cell damage [10]. UPR is indeed a stress response pathway that promotes the inflammatory response and plays a critical role in a wide range of cellular pathologies. In addition, several studies have described the involvement of UPR in bacterial infection [25]. In those reports, UPR was closely related to cytotoxicity, the inflammatory response, and apoptosis of host cells in response to bacterial infection. Moreover, during invasive bacterial infection, induction of UPR was remarkable in response to *Listeria monocytogenes* [26], *Legionella pneumophila* [27], and *Mycobacterium tuberculosis* [28]. These studies also reported that ER stress functions as an innate defense mechanism against intracellular bacterial invasion.

Despite the strong association between the invasion of various bacterial species and ER stress, such relationships between ER stress and *C*. *jejuni* infection remain unclear. Therefore, the aim of the present study was to advance the understanding of the modulation of host cell signaling in response to *C*. *jejuni* infection by investigating the role of UPR in the infection process. The data showed that UPR was activated by infection with *C*. *jejuni* and that treatment with the ER stress inducers thapsigargin and tunicamycin inhibited the ability of *C*. *jejuni* to invade Caco-2 cells. Also, ER stress induced by pre-infection with *C*. *jejuni* decreased the extent of subsequent *C*. *jejuni* invasion. Furthermore, suppression of UPR-signaling proteins facilitated *C*. *jejuni* infection of HeLa cells. These results suggest that UPR is activated as a defense strategy against *C*. *jejuni* infection.

## Material and methods

### Bacterial strains and culture conditions

*C*. *jejuni* strains NCTC11168 (ATCC 700819) and 81–176 (ATCC BAA2151) were obtained from the American Type Culture Collection (Manassas, VA, USA). Wild-type (WT) *C*. *jejuni* strain NCTC11168 was used as a standard strain in this study. The bacteria were grown in Mueller–Hinton broth (MHB; DIFCO 275730; Difco Laboratories, Franklin Lakes, NJ, USA) under a microaerobic atmosphere (5% O_2_, 10% CO_2_, 85% N_2_) at 37°C for 48 h. The bacteria cells were centrifuged at 12,000 rpm for 3 min and concentrated. Then, a 20-μL aliquot of the bacterial suspension was injected into fresh MHB and grown under a microaerobic atmosphere at 37°C for an additional 36 h. *S*. *enterica* serovar enteritidis (*S*. Enteritidis) was cultured in Luria-Bertani (LB) medium at 37°C with shaking.

### Construction of nalidixic acid (NA)-resistant *C*. *jejuni*

WT *C*. *jejuni* (NCTC11168) was grown in MHB, concentrated to an optical density at 600 nm (OD_600_) of 1.0 with phosphate-buffered saline (PBS), plated on MH ager containing 100 μg/mL of NA, and incubated under a microaerobic atmosphere at 37°C for 48 h. Then, colonies were picked and grown in NA containing MHB for 48 h. Genomic DNA was purified and the gyrA fragment (849 bp) was amplified by polymerase chain reaction (PCR) with the indicated primers (Table 1). The gyrA fragment was sequenced to verify the presence of a G to A substitution at gene position 371 [29]. The bacterial strains used in this article are listed in Table 1.

**Table 1:** *C*. *jejuni* strain list.

*C*. *jejuni* strain	Discription	Referense
**wild type**		
NCTC11168	Wild-type strain	-
81–176	Wild-type strain	-
**mutant**		
gyrA G371A	Nalidixic acid resistant	[29]
CapA::Cm^r^	Adhesion and invasion deficient	[30]
CadF::Cm^r^	Adhesion and invasion deficient	[31]
Cj0268::Cm^r^	Adhesion and invasion deficient	[32]

### Cell culture

All of cell lines were purchased from ATCC. Human epithelial colorectal adenocarcinoma (Caco-2) cells, human intestinal cell line (INT407) cells and human colon adenocarcinoma (HT-29) cells were cultured in Dulbecco's modified Eagle's medium (DMEM; Sigma-Aldrich Corporation, St. Louis, MO, USA) supplemented with 10% fetal bovine serum (FBS; Gibco BRL, Carlsbad, CA, USA) and 100 mg/mL of gentamycin (Sigma-Aldrich Corporation), referred to as DMEM-FBS(+) hereafter. The cells were seeded in the wells of 6-well culture dishes at a density of 3×10^5^ cells/well and cultured for 4 days at 37°C under a humidified atmosphere of 5% CO_2_/95% air. Human epithelioid cervix carcinoma HeLa cells were cultured in DMEM containing 10% FBS (Thermo Fisher Scientific, Waltham, MA, USA) and 100 mg/mL of gentamycin. Then, the cells seeded in the wells of 6-well culture dishes at a density of 3×10^5^ cells/well and cultured for 3 days at 37°C under a humidified atmosphere of 5% CO_2_/95% air.

### Construction of a reporter system with an enhanced green fluorescent protein (eGFP) plasmid

A hemagglutinin (HA)-tagged X-box binding protein-1 (XBP1) fragment was constructed by PCR so that both sequences included a Kozak sequence and HA-tag coding sequence along with 409–633 bp of the human XBP1 cDNA. HA-XBP1-EGFP was generated by overlapping PCR and cloned into the mammalian expression vector pcDNA 3.1 (+). The nucleotide arrangement of the XBP1 fragment is reported elsewhere [33]. The plasmid pcDNA-HAXBP1-EGFP was used as an indicator in cultured Caco-2 cells.

### Reagents

The ER stress inducers thapsigargin and tunicamycin were obtained from Wako Pure Chemical Industries, Ltd. (Osaka, Japan) and Sigma-Aldrich Corporation, respectively. MβCD, chlorpromazine, and colchicine were purchased from Sigma-Aldrich Corporation and dissolved in dimethyl sulfoxide (DMSO) as stock solutions.

### Infection protocol

The culture medium for Caco-2 cells was replaced with fresh DMEM medium without supplements (DMEM-FBS(-)) at least 3 h before infection. *C*. *jejuni* were centrifuged at 3,000 rpm for 15 min, washed with PBS (pH 7.4), and then adjusted to a concentration of OD_600_ = 1.0 with PBS. *S*. Enteritidis were harvested by centrifugation at 12000 rpm for 3 min, washed, and adjusted with PBS similar to *C*. *jeuni*. Cells were infected at a multiplicity of infection (MOI) of 100–200:1 in *C*. *jejuni* infection or 10–20:1 in *S*. Enteritidis infection. The cells were infected with bacteria at a multiplicity of infection of 100 at 37°C under an atmosphere of 5% CO_2_/95% air.

### Transcriptional analyses

Total mRNA was isolated from Caco-2 cells at 6 and 12 h after infection with TRIzol reagent (Invitrogen Corporation, Carlsbad, CA, USA), treated with RNase-free DNAase I (TaKaRa Biotechnology (Dalian) Co., Ltd., Dalian, China), and then reverse transcribed into cDNA using the PrimeScript RT-Reagent Kit (TaKaRa). Each reaction mixture contained 500 ng/10 μL of RNA, 0.5 mL/l0 μL of random hexamers (Thermo Fisher Scientific), and 0.5 mL/10 μL of oligo nucleoside triphosphates. Quantitative real-time reverse-transcription PCR (qPCR) was performed using the LightCycler Real-Time PCR System (Roche Applied Science, Penzberg, Germany) in a volume of 12 μL with SYBER Premix Ex Taq (TaKaRa). Each reaction contained 1.2 μL of cDNA and 0.24 μL of primers. Endogenous glyceraldehyde 3-phosphate dehydrogenase (GAPDH) mRNA was used for normalization of the transcriptional levels of CHOP and GADD34. The human-specific primers shown in Table 2 were used for qPCR (“F” and “R” indicate forward and reverse, respectively).

**Table 2:** Primers list.

No.	Target		Primer sequence (5’–3’)
**1**	*C*. *jejuni* gyrA	F	GGTTCTAGCCTTTTGGAAGC
		R	CGCCCTGTGCGATAAGCTTC
**2**	18S	F	AAACGGCTACCACATCCAAG
		R	GGCCTCGAAAGAGTCCTGTA
**3**	CHOP	F	GCACCTCCCAGAGCCCTCACTCC
		R	GTCTACTCCAAGCCTTCCCCCTGCG
**4**	GADD34	F	ATGTATGGTGAGCGAGAGGC
		R	GCAGTGTCCTTATCAGAAGGC
**5**	PERK	F	CCTGCTTCTACAGCGTACCC
		R	TACCGAAGTTCAAAGTGGCC
**6**	IRE1	F	CGGCCTTTGCAGATAGTCTC
		R	GTCAGATAGCGCAGGGTCTC
**7**	ATF6	F	GCAGAACCTCAGCCACTTC
		R	TGTGGAACACTGGAGTTTG

### Protein preparation from eukaryotic cells

At the end of the infection period, the medium was removed, and then the cells were washed once with ice-cold PBS and then scraped into radioimmunoprecipitation assay buffer (50 mM Tris-HCl, pH 7.4, 150 mM NaCl, 1% Triton X-100, 1% sodium deoxycholate, and 0.1% sodium dodecyl sulfate [SDS]) containing 10% protease inhibitor mix. Cell lysates were homogenized using a needle and syringe, then cleared by centrifugation at 14,800 rpm for 10 min at 4°C. Total protein in the supernatants was quantified using a BCA Protein Assay Kit (Pierce Biotechnology, Waltham, MA, USA) and stored at −80°C.

### Western blotting

The cell lysates were mixed with sample buffer (pH 6.8, 100 mM Tris, 25% glycerol, 2% SDS, 0.01% bromophenol blue, 10% 2-mercaptoethanol) and boiled for 5 min at 95°C. Equal amounts of protein were loaded on SDS–polyacrylamide gels, separated by electrophoresis, and then transferred to polyvinylidene difluoride membranes, which were blocked with blocking buffer (3% milk in TBS with 0.1% Tween-20) for 1 h at room temperature and incubated with primary antibodies overnight at 4°C. Afterward, the membranes were washed 3 times with TBS-T, incubated with immunoglobulin (Ig)G-horseradish peroxidase-conjugated secondary antibodies for 2 h at room temperature, washed 3 times with TBS-T 3, and visualized with the enhanced chemiluminescence (ECL) Western Blotting Kit (GE Healthcare Bio-Sciences Corp., Piscataway, NJ, USA). Antibodies against eIF2α, phospho-eIF2α (Ser51), p70 S6Kinase, phosphor-p70 S6Kinase (Thr389), ATF6, and CHOP were purchased from Cell Signaling Technology, Inc. (Beverly, MA, USA). Anti-GFP antibody was from Abcam (Cambridge, UK). Antibodies against GAPDH and β-actin were from Santa Cruz Biotechnology, Inc. (Dallas, TX, USA). Anti-puromycin antibody (#2856058) was from EMD Millipore Corporation (Billerica, MA, USA). Anti-tubulin antibody was from Wako Pure Chemical Industries, Ltd. Horseradish peroxidase-conjugated goat anti-rabbit IgG, goat anti-mouse IgG, and anti-goat IgG were from Medical & Biological Laboratories Co., Ltd. (Nagoya, Japan).

### Puromycin incorporation assay

After infection, the cells were treated with 10 μg/mL of puromycin for 30 min, washed 3 times with ice-cold PBS, collected, and then detected by Western blotting with puromycin-specific antibodies.

### shRNA knockdown

The pENTR^TM^/U6 vector (Invitrogen Corporation) was used for PERK, IRE1, and ATF6 knockdown assays. The target sequences of short-hairpin RNAs (shRNAs) against PERK (5’-tagtgacgaaatggaacaaga-3’), IRE1 (5’-agcaggacatctggtatgtta-3’), ATF6 (5’-cagcaaatgagacgtatgaaa-3’), and lacZ (double-stranded control oligos) were cloned into the pENTR^TM^/U6 vector. HeLa cells were seeded at a density of 2.5 × 10^5^ cells/well in 24-well plates and cultured for 1 day. The medium was replaced with DMEM-FBS(−) and transfected with pENTR-PERK, pENTR-IRE1, pENTR-ATF6, or the control vector using Lipofectamine 2000 reagent (Invitrogen Corporation), respectively. After transfection for 3 h, FBS and gentamycin were added to the medium, and the cells were cultured for an additional 2 days.

### Invasion, adhesion, and survival assay

*C*. *jejuni* invasion of host cells was measured with a gentamycin protection assay. Briefly, Caco-2 cells were cultured in the wells of 24-well plates for 4 days. The culture medium was replaced with DMEM-FBS(-) containing thapsigargin or tunicamycin and the cells were incubated at 37°C under a humidified atmosphere of 5% CO_2_ for 12 h. After 12 h, the cells were washed 1 time with DMEM-FBS(-), and the medium was replaced with DMEM-FBS(-). Then, the cells were infected with *C*. *jejuni* for 3 h. After infection, the cells were incubated with DMEM-FBS(-) containing 100 μg/mL of gentamycin for 2 h to remove extracellular bacteria. Afterward, the cells were washed once with PBS and lysed with PBS containing 1% Triton-X for 5 min at 37°C. The cell lysates containing bacteria were plated on MH agar and incubated for 48 h at 37°C under a microaerobic atmosphere. The quantity of intercellular bacteria was estimated by counting colony-forming units.

In adhesion assays, after 1 h infection, supernatants were removed and cells were washed three times with PBS before lysis with PBS containing 1% Triton X at 37°C for 5 min, and cell lysates were plated on MH agar plates and incubated for 48 h under microaerobic atmosphere.

In survival assays, after 3 h infection, supernatants were removed and replaced with DMEM-FBS(−) containing 100 μg/ml of gentamycin for 5 h in Caco-2 cells, or 6 h in shRNA transfected HeLa cells. Cells were lysed with PBS containing 1% Triton-X, and cell lysates were plated on MH agar plates and incubated for 48 h under microaerobic atmosphere.

### Quantification of NA-resistant *C*. *jejuni* invasion

Caco-2 cells were cultured in the wells of 24-well culture dishes for 4 days. Then, the cells were infected with WT *C*. *jejuni* for 12 h, washed 3 times with DMEM-FBS(-), and infected with NA-resistant *C*. *jejuni* for 6 h. The cell lysates, collected as described above, were plated on MH agar plates containing 25 μg/mL of NA and incubated for 48 h at 37°C under a microaerobic atmosphere. The quantity of intercellular bacteria was evaluated by counting colony-forming units.

### Uptake of FITC-Dextran

Cells were cultured on 24-well plates and stimulated with ER stress inducers or induced shRNA knockdown. Cells were washed with PBS and incubated with DMEM-FBS(−) containing 0.1 mg/ml 10 kDa FITC-dextran for 2 h. After incubation, the cells were washed with PBS three times and lysed with RIPA buffer. Cell lysates were centrifuged at 15,000 rpm for 10 min, and supernatant was collected. The supernatants were measured using a fluorescence microplate reader (Molecular Devices) at excitation and emission wavelengths of 490 and 520 nm, respectively.

## Results

### *C*. *jejuni* infection suppressed the global protein translation in Caco-2 cells

**Fig 1:**
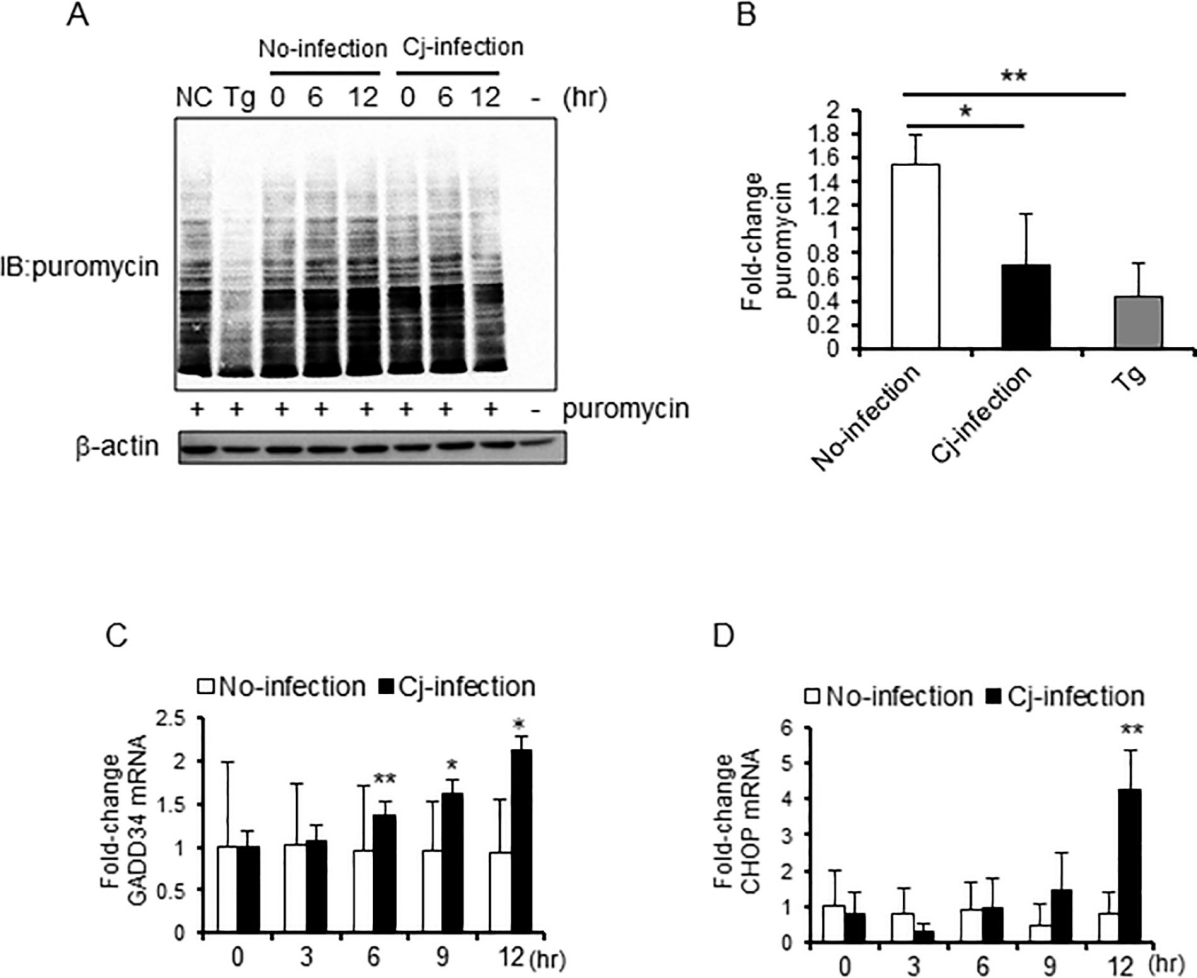
Intracellular global protein translation was suppressed during *C. jejuni* infection. (A and B) Caco-2 cells were infected with *C*. *jejuni* for the indicated times, treated with puromycin (10 μg/mL), and incubated at 37°C for 30 min before being collected. The incorporation of puromycin was detected by western blotting. β-actin was used as an internal control for protein loading. (B) Each lane was quantitated, and the total puromycin levels were indicated by relative values, vs. no-infection group at 0 h. Puromycin levels were normalized by β-action expression levels. (C and D) Caco-2 cells were treated with PBS or infected with *C*. *jejuni*, and RNA was isolated at the indicated times. qPCR was performed to detect CHOP and GADD34 transcript levels. Each mRNA was with all values normalized to the β-actin housekeeping gene. Asterisks denote significant differences (**p < 0.01, by t-test, n = 4–6). Each experiment was repeated three times (A was repeated four times).

UPR signaling is related to inhibition of global protein translation to prevent the accumulation of unfolded proteins. First, to determine whether *C*. *jejuni* infection inhibits global protein translation, puromycin incorporation assay of intestinal cultured Caco-2 cells was conducted. The translational products were detected with puromycin-specific antibody, which showed that the global protein translation in *C*. *jejuni*-infected cells had decreased (Fig 1A and 1B). Furthermore, during *C*. *jejuni* infection, mRNA expression levels of ER-associated transcription factors, such as CHOP and growth arrest and DNA damage-inducible protein-34 (GADD34), were up-regulated after infection for 12 h (Fig 1C and 1D). These data suggest that unfolded proteins had accumulated, and ER stress was induced by *C*. *jejuni* infection.

### UPR was induced by *C*. *jejuni* infection

**Fig 2:**
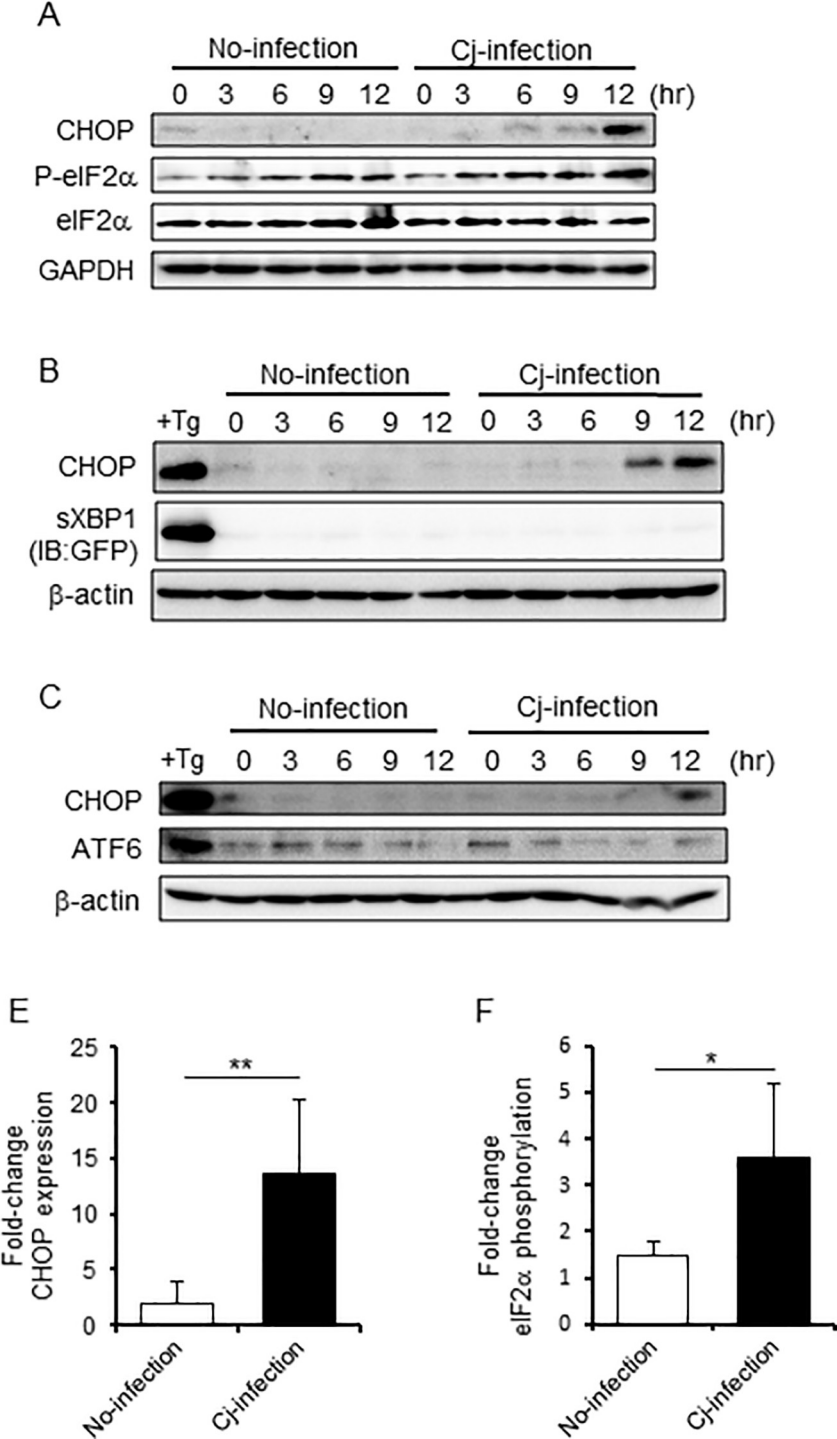
UPR was activated by *C*. *jejuni* infection. (A-C) Western blotting of UPR signaling proteins, including phosphorylated eIF2α (Ser51), total eIF2α, ATF6, and CHOP, in lysates of Caco-2 cells treated with PBS or infected with *C*. *jejuni* was conducted at 3-h intervals for a 12-h period (A, C). Spliced XBP1 was detected by GFP expression. As a positive control, cells were treated with 2.5 μM thapsigargin, an inducer of ER stress, for 12 h. (D and E) Quantification of the relative CHOP expression and eIF2α phosphorylation levels were indicated by relative values, vs. no-infection group at 0 h. CHOP was normalized to the α-tubulin housekeeping protein expression, and phosphorylated-eIF2α was normalized to the total protein expression of eIF2α. Asterisks denote significant differences (*p < 0.05, **p < 0.01, by t-test, n = 5). Each experiment was repeated three times (A was repeated five times).

To advance the understanding of how *C*. *jejuni* infection induces ER stress, changes to UPR signaling during *C*. *jejuni* infection were observed. UPR sensor protein signaling was categorized according to the involvement of PERK, ATF6, and IRE1. Activation of PERK led to phosphorylation of the α-subunit of eukaryotic translation initiation factor-2 (eIF2α) at Ser51. IRE1 led to splicing of X-box binding protein-1 (xbp1) mRNA [34–36]. In response to ER stress, ATF6 is translocated to the Golgi body, resulting in its cleavage by site-1 and site-2 proteases [19, 21, 24, 37]. For analysis of these three pathways, the phosphorylation of eIF2α, the splicing XBP1 (XBP1s), and the cleavage of ATF6 were estimated by Western blotting. In addition, protein expression of CHOP, a downstream translation factor of these pathways, was also checked as a marker of ER stress. With the increase in mRNA levels (Fig 1C), the expression of CHOP protein was up-regulated in host cells infected with *C*. *jejuni*. Similar to CHOP expression, the amount of phosphorylated eIF2α was also increased, but spliced XBP1s and cleaved ATF6 were not detected in *C*. *jejuni*-infected cells (Fig 2A–2C). Also, infection with *C*. *jejuni* strain 81–176 produced similar results in regard to ER stress sensor signaling (S1A Fig). Then, the increases in eIF2α phosphorylation and CHOP in HeLa cells and HT-29 cells were confirmed (S1B and S1C Fig).

### UPR was induced in a manner independent of *C*. *jejuni* intracellular invasion

**Fig 3:**
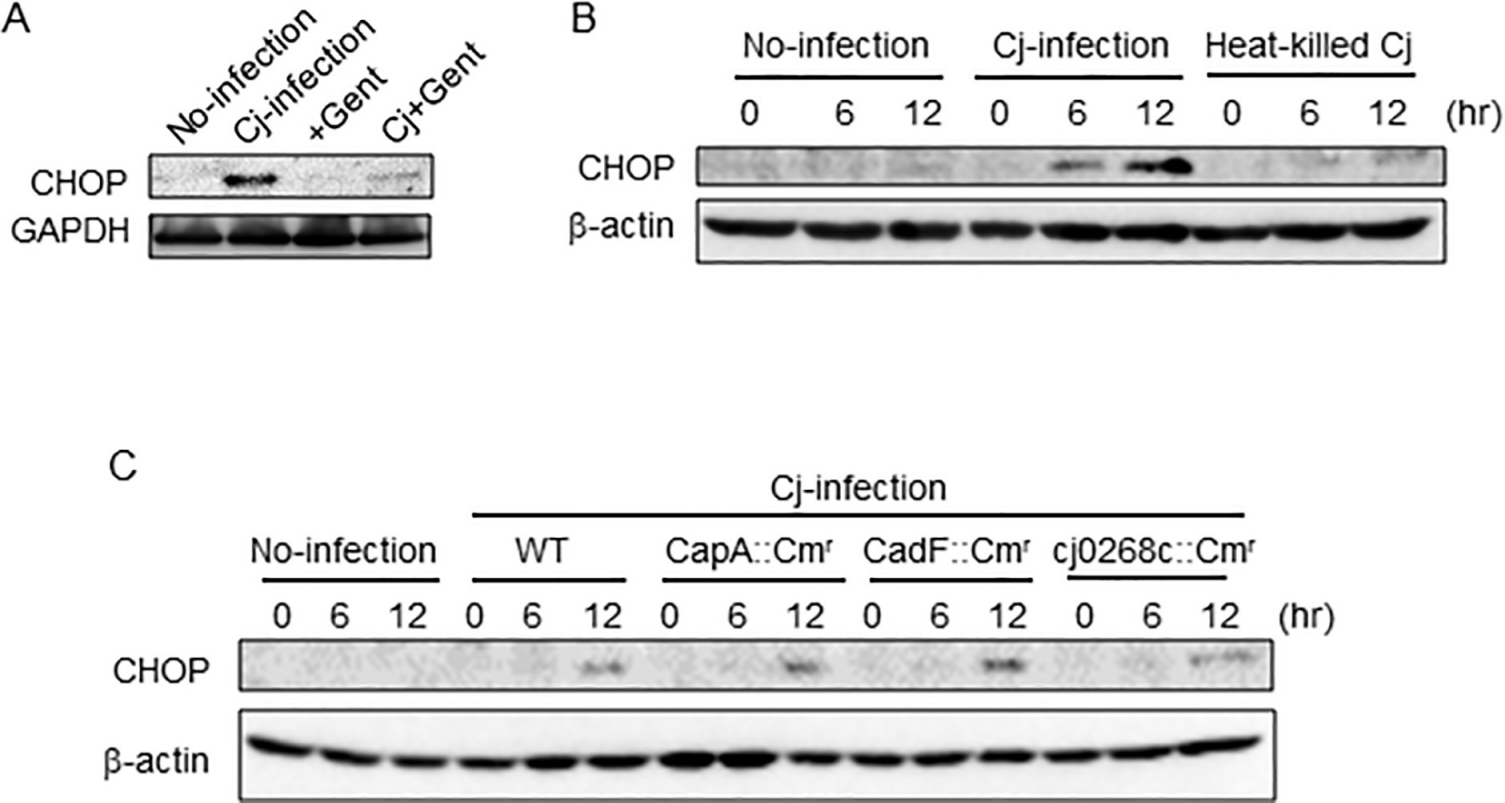
UPR was induced independently of *C*. *jejuni* invasion. (A) Caco-2 cells were infected with *C*. *jejuni* or treated with only gentamycin, *C*. *jejuni* and gentamycin, and collected after 12 h. (B) Caco-2 cells were treated with PBS, heat-killed (at 95°C for 10 min) *C*. *jejuni* or infected with no-treatment *C*. *jejuni*. (C) Cells were infected with adhesion and invasion deficient strains, CapA::Cm^r^, CadF::Cm^r^, and 0268::Cm^r^ collected at indicated times. CHOP expression was detected by Western blotting. β-actin was used as internal control for protein loading. Each experiment was repeated three times.

Next, we estimated the participation of bacterial invasion in UPR induction. The activation of UPR was not observed in gentamycin-treated or heat-killed *C*. *jejuni* (Fig 3A and 3B). Also, we checked invasive effect using invasion decreasing mutant strains (CapA::Cm^r,^ CadF::Cm^r^, and cj0268c::Cm^r^). In the previous report, the reduction of invasion was confirmed [30–32]. UPR was activated by the adhesion or invasion-deficient mutant *C*. *jejuni* strain CapA::Cm^r^, CadF::Cm^r^, and cj0268c::Cm^r^ infected Caco-2 cells (Fig 3C). Taken together, these results indicate that *C*. *jejuni* infection induces UPR, suggesting that UPR is induced by living *C*. *jejuni*, and intracellular invasion did not trigger the induction of UPR.

### UPR signaling plays an important role in host defense against *C*. *jejuni* infection

**Fig 4:**
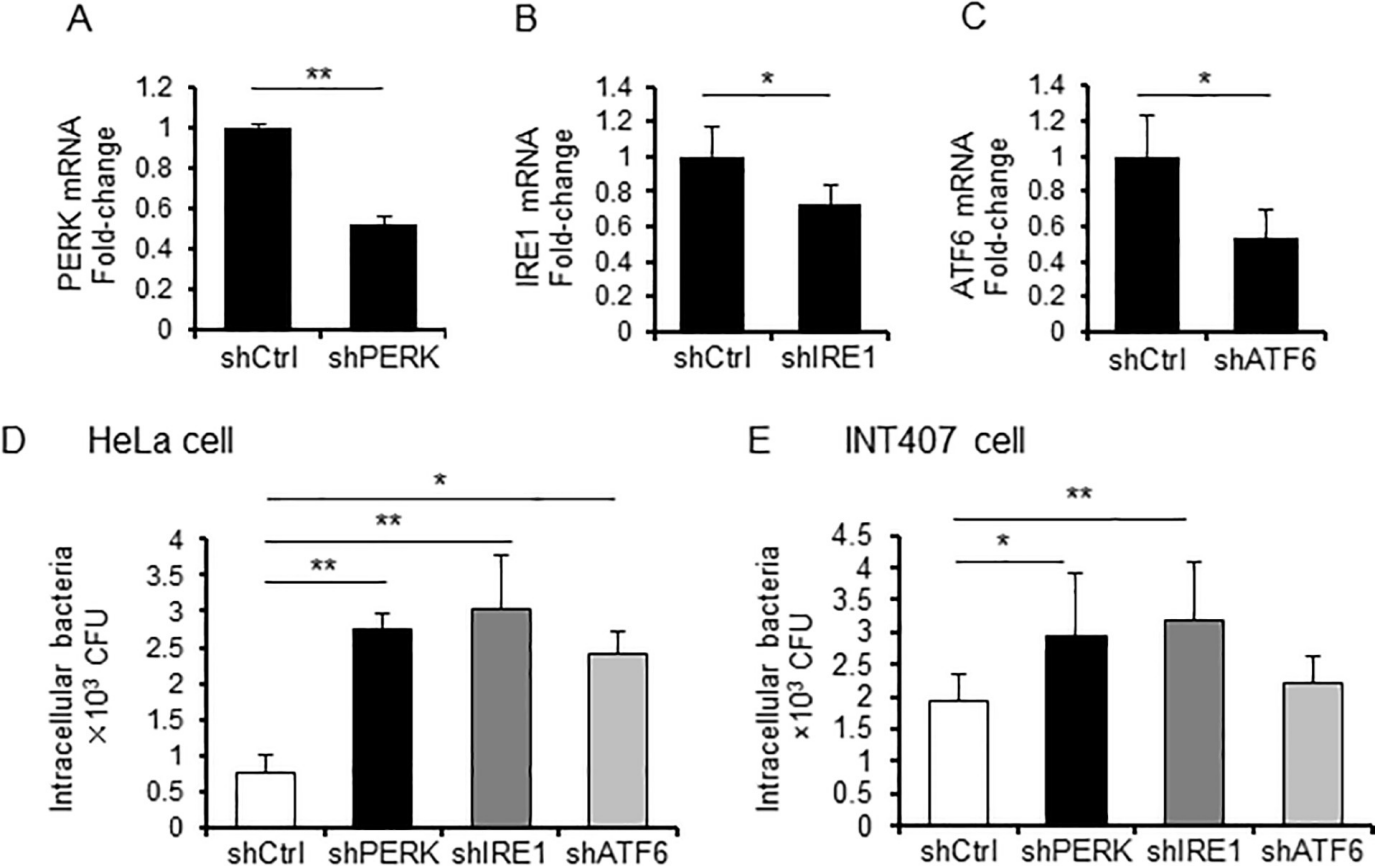
UPR signaling plays an important role in the defense response against *C*. *jejuni* infection. (A-C) HeLa cells were transfected with the pENTR-PERK vector, the pENTR-IRE1 vector, the pENTR-ATF6 vector, or a non-target shRNA control vector, and transcriptional levels of each target were analyzed using qPCR. Each mRNA was normalized to the housekeeping gene β-actin. (D and E) HeLa and INT407 cells, transfected with pENTR-PERK vector, pENTR-IRE1 vector pENTR-ATF6 vector, or non-target shRNA control vector, were infected by *C*. *jejuni* for 3 h, and intracellular bacteria numbers were estimated with a gentamicin protection assay (*p < 0.05, **p < 0.01, by t-test, n = 3–6). Each experiment was repeated three times (D was repeated four times).

To investigate the effect of UPR signaling on bacterial invasion of host cells, which plays an important role in *C*. *jejuni* pathogenicity, was evaluated. The intracellular invasion of *C*. *jejuni* was examined using PERK-, IRE1-, or ATF6-targeted shRNA-transfected HeLa cells. As shown in Fig 4A–4C, the transcriptional levels of PERK, IRE1, and ATF6 were decreased in the shRNA-transfected cells. The intercellular invasion of *C*. *jejuni* was increased in PERK-, IRE1-, and ATF6-knockdown cells, as compared with cells infected with the sh-control vector (Fig 4D). An increase in intracellular invasion of *C*. *jejuni* was observed in INT407 cells (Fig 4E). These results indicate that UPR signaling is induced as a defense mechanism against *C*. *jejuni* infection.

### *C*. *jejuni*-induced ER stress influences intercellular bacterial invasion

**Fig 5:**
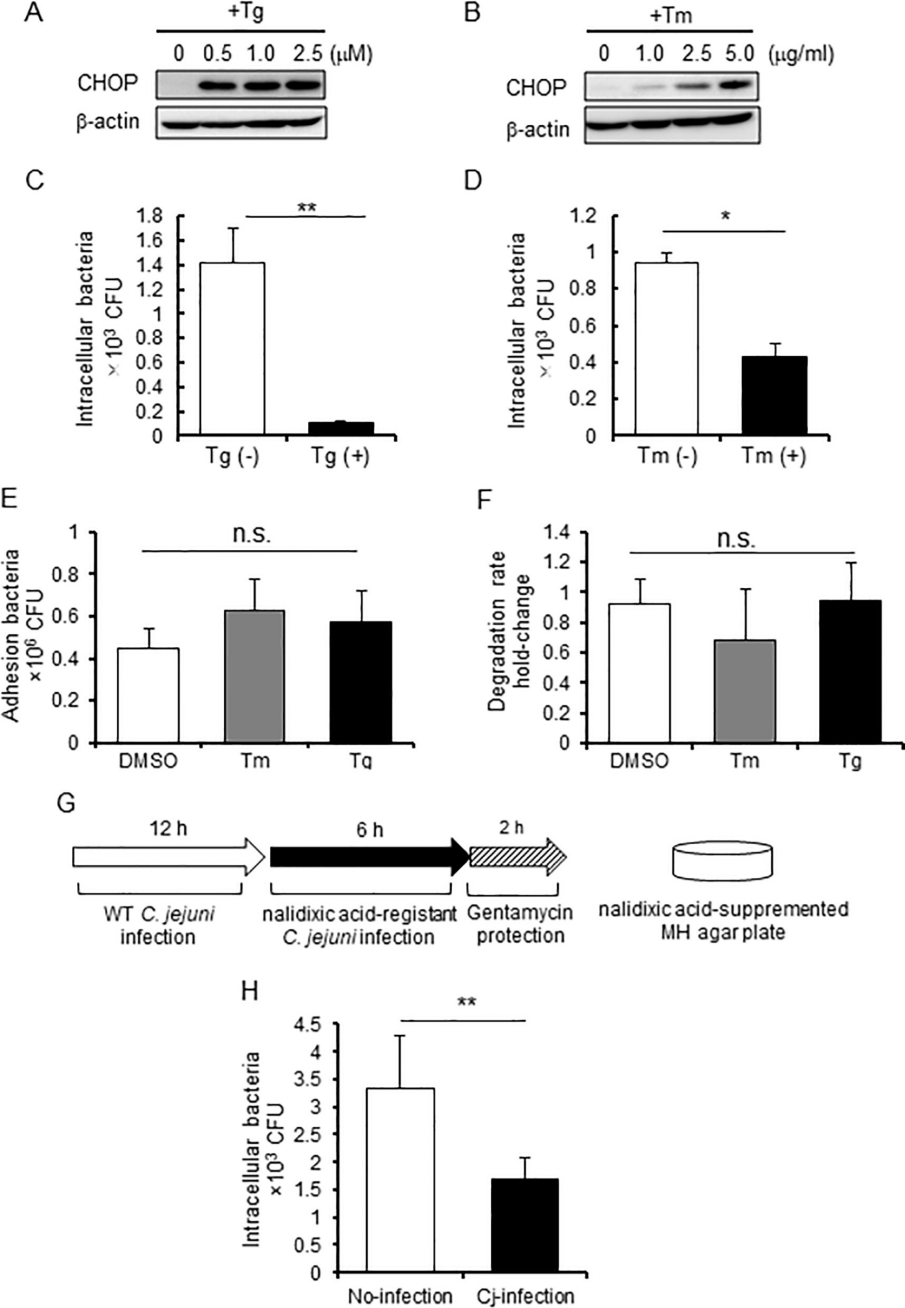
ER stress attenuated *C*. *jejuni* invasion. (A and B) Caco-2 cells were treated with thapsigargin (0, 1.0, 2.5, or 5.0 μM) or tunicamycin (0, 1.0, 2.5, or 5.0 μg/mL) and collected after 12 h. UPR signaling was detected by Western blotting. (C-F) Caco-2 cells were pretreated with thapsigargin (2.5 μM), tunicamycin (5.0 μg/mL), or DMSO (as a control) for 12 h and infected with *C*. *jejuni* for 6 h. Then, bacterial invasion (C and D), adhesion (E), and intracellular survival (F) were assessed after 1 or 3 h using the gentamycin protection assay. (G and H) A selective assay of intracellular NA-resistant *C*. *jejuni* was performed. Caco-2 cells were infected with WT *C*. *jejuni* for 12 h, then washed with DMEM-FBS(-) three times to eliminate extracellular bacteria. After pre-infection, the cells were infected with NA-resistant *C*. *jejuni* for 6 h, and the number of intracellular NA-resistant bacteria was selectively estimated using the gentamycin protection assay. Asterisks denote significant differences (n.s., not significant, *p < 0.05, **p < 0.01, by t-test, n = 4). Each experiment was repeated four times.

Next, the effect of ER stress on bacterial invasion of host cells was investigated. Briefly, Caco-2 cells were treated with two types of ER stress inducers: thapsigargin, which inhibits sarcoplasmic/ER Ca^2+^ ATPase and induces ER stress by depleting Ca^2+^ ions from the ER, and tunicamycin, which blocks protein glycosylation in the ER and induces ER stress. The activation of UPR signaling by these inducers was confirmed using Western blotting and by observing CHOP expression (Fig 5A and 5B). Next, the *C*. *jejuni* invasion level in cells pretreated with thapsigargin or tunicamycin was investigated. As shown in Fig 5C and 5D, the number of intracellular *C*. *jejuni* was decreased in these cells. It suggested that the number of intracellular bacterial was affected at several steps during infection, such as adhesion, invasion, and intracellular survival. We next analyzed whether thapsigargin and tunicamycin have an effect on *C*. *jejuni* adhesion or intracellular degradation, so that adhesion or degradation were not changed in cells pretreated with these ER stress inducers. These results suggest that ER stress suppresses intracellular invasion of *C*. *jejuni*. Furthermore, the influence of UPR induction during *C*. *jejuni* infection to *C*. *jejuni* invasion was examined. As shown the scheme in Fig 5G, UPR was induced by *C*. *jejuni* pre-infection for 12 h then the cells were infected with NA-resistant *C*. *jejuni*. In order to selectively count the intracellular NA-resistant *C*. *jejuni* number, cells were plated on MH agar containing NA (Fig 5G). As a result, there was a significant reduction in the number of intracellular NA-resistant bacteria after pre-infection with WT *C*. *jejuni*, as compared to non-pre-infection (Fig 5H). These data raise the possibility that UPR also participated in the regulation of bacterial invasion during *C*. *jejuni*-infection.

### Decrease in the invading bacterial number caused by UPR specifically occurs in *C*. *jejuni*

**Fig 6:**
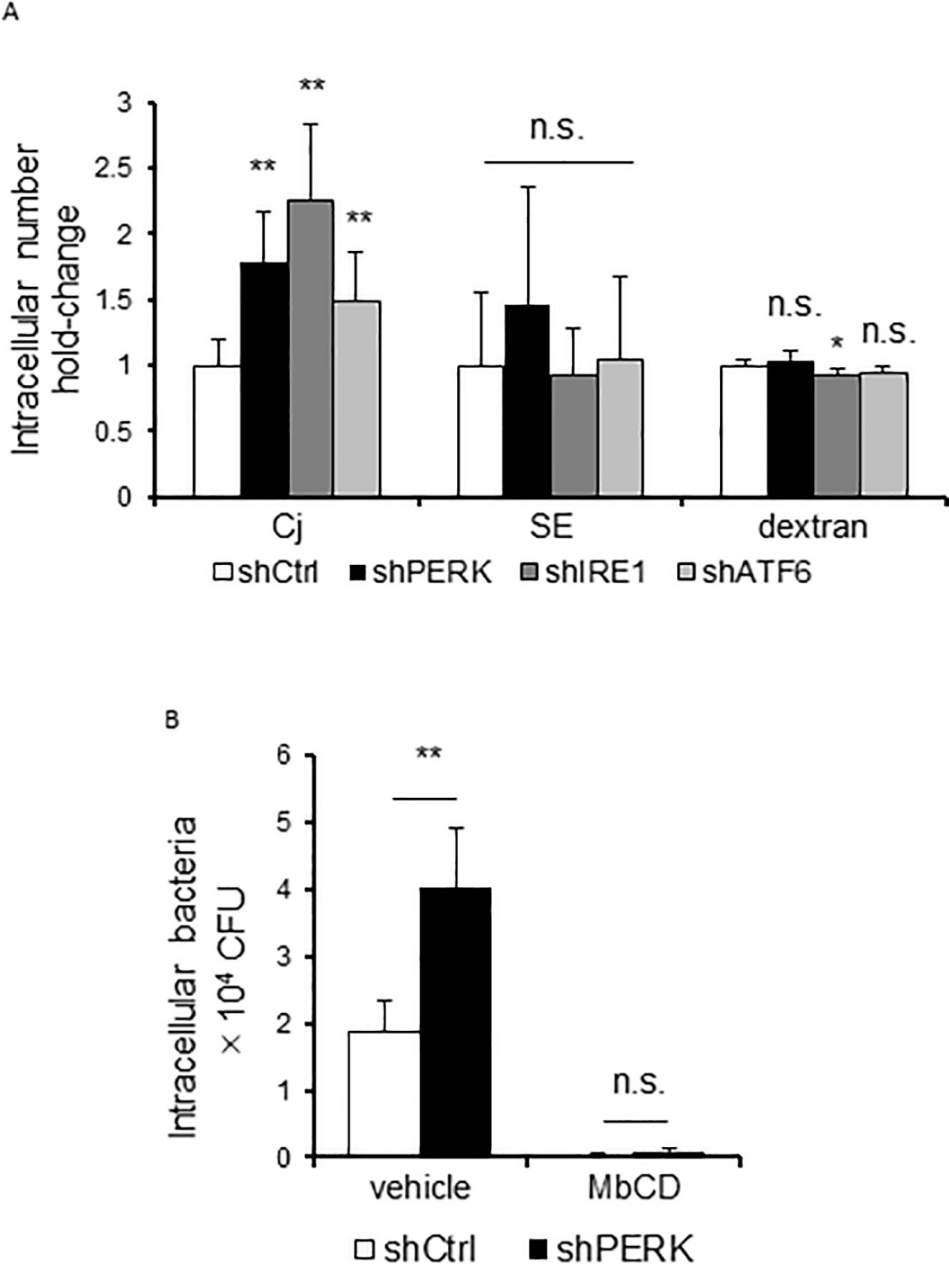
The effect of UPR on intracellular intake is specific to *C*. *jejuni* infection. (A) HeLa cells transfected with pENTR-PERK vector, pENTR-IRE1 vector pENTR-ATF6 vector, or non-target shRNA control vector were infected by *C*. *jejuni* for 3 h or by *S*. Enteritidis for 1 h or treated with 0.1 mg/mL 10 kDa FITC-dextran for 2 h. After infection, intracellular bacteria numbers were estimated using a gentamicin protection assay. FITC-dextran uptake was measured by fluorescence. (B) HeLa cells transfected with pENTR-PERK vector or non-target shRNA control vector were treated with MβCD (7.5 mM) for 1 h, the number of intracellular bacteria was estimated using the gentamicin protection assay. Asterisks denote significant differences versus control groups (n.s., not significant, *p < 0.05, **p < 0.01, by t-test, n = 6). Each experiment was repeated three times.

To investigate whether UPR-mediated suppression of bacterial invasion was specific to *C*. *jejuni*, we examined the effects of UPR on the invasion of another invasible pathogen, *S*. Enteritidis. Unlike *C*. *jejuni*, *S*. Enteritidis invasion was not promoted in UPR signaling-knockdown HeLa cells (Fig 6A). Further, treatment with ER stress inducers, thapsigargin and tunicamycin, did not suppress *S*. Enteritidis invasion in Caco-2 cells (S3 Fig). It has been indicated that *C*. *jejuni* is internalizing via the endocytosis-like uptake pathway [38]. Next, we analyzed the influence of UPR on endocytosis pathway by evaluating the uptake of FITC-dextran. UPR signaling-knockdown or ER stress inducers treated cells indicated different uptake between FITC-dextran and *C*. *jejuni* bacterial cells (Fig 6A and S3 Fig). These results suggest that UPR shows the decreasing of *C*. *jejuni*-invasion specifically. In the *C*. *jejuni* invasion mechanisms, lipid rafts, which are abundant in cholesterol and sphingolipid plasma membrane microdomain, were essential for *C*. *jejuni* entry via the caveolae mediated endocytosis pathway [39, 40]. Therefore, we focused on lipid rafts-mediated endocytosis as the mechanism of *C*. *jejuni* invasion. The components of lipid rafts, such as cholesterol, are removed by treatment with MβCD [41]; the number of invading *C*. *jejuni* cells was decreased by the addition of MβCD. In addition, treatment with MβCD cancelled the difference in the number of intracellular bacteria between control cells and PERK knockdown cells (Fig 6C). These results suggested that UPR signaling may affect the distribution or expression of *C*. *jejuni* invasion targeting factors into the lipid-rafts mediated invasion pathway.

## Discussion

Infection with some bacterial species induces ER stress, which is characterized by the accumulation of unfolded proteins in the ER and perturbation of ER homeostasis. In response to ER stress, UPR signaling is induced and plays a key role in the maintenance of protein homeostasis. A previous study revealed that efficient intracellular invasion of *C*. *jejuni* was closely associated with the release of Ca^2+^ from host stores in the ER [42]. This report raises the possibility that *C*. *jejuni* infection affects ER homeostasis.

**Fig 7:**
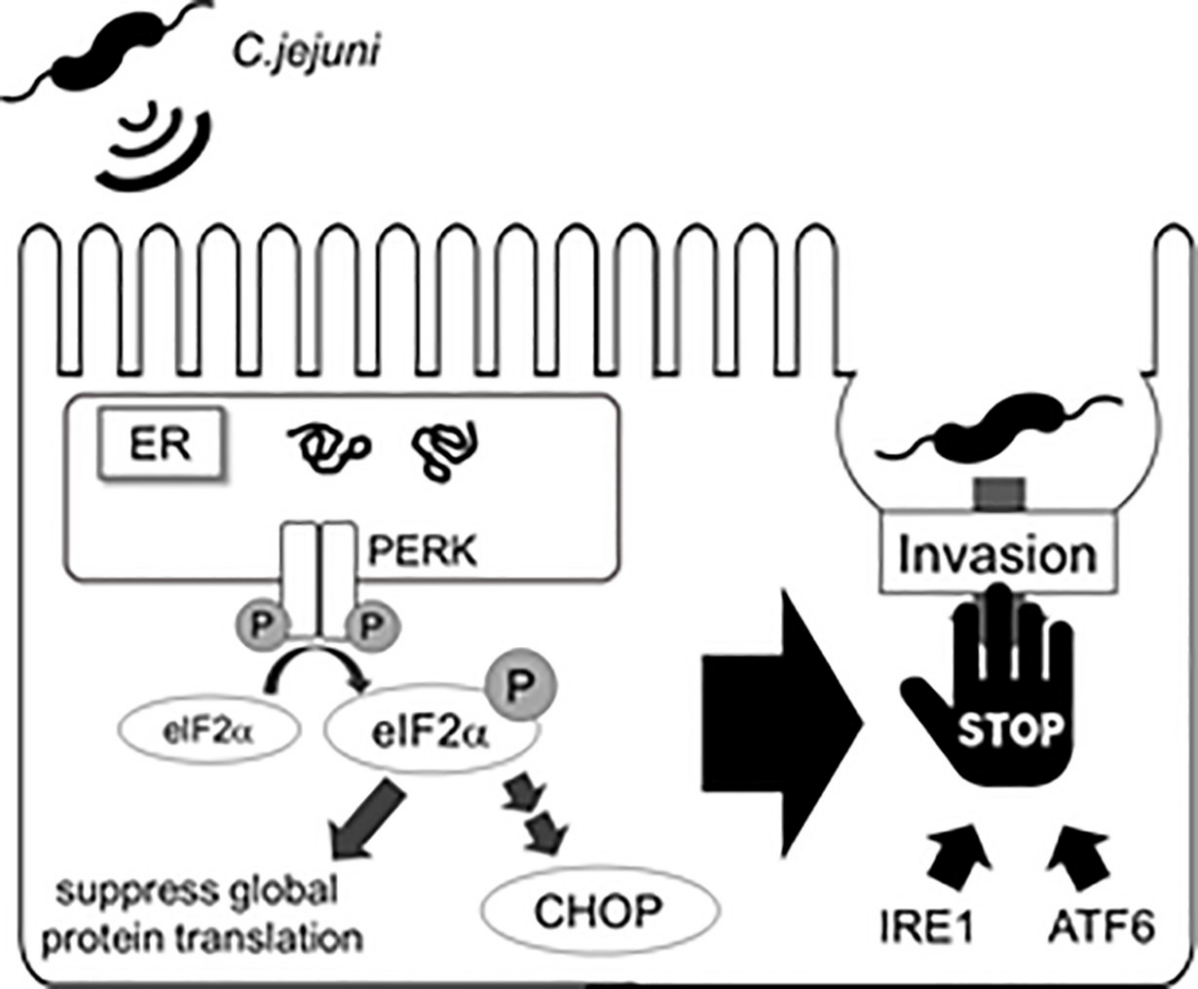
Comprehensive model of UPR induction during *C*. *jejuni*. UPR signaling induced in *C*. *jejuni* infected cells leads to suppress global protein translation and invasion of *C*. *jejuni* into the host cells.

We hypothesized that *C*. *jejuni* modulate the host cell signaling and induces ER stress, and the stress response may affect bacterial virulence, such as adhesion or invasion. To investigate this hypothesis, the degree of stress induced by UPR signaling in *C*. *jejuni*-infected cells was estimated. The results showed that *C*. *jejuni* infection decreased global protein translation (Fig 1A and 1B) and increased the expression levels of ER stress-related factors, including CHOP and GADD34 (Fig 1C). Moreover, eIF2α-phosphorylation and CHOP expression were induced in *C*. *jejuni*-infected Caco-2 cells. These data strongly suggest that ER stress was provoked by *C*. *jejuni* infection and UPR signaling was activated to maintain protein homeostasis in response to stress induced by infection. In order to elucidate the role of UPR signaling activation in *C*. *jejuni* infection, the contribution of UPR signaling in *C*. *jejuni* invasion was investigated using PERK, IRE1, and ATF6 shRNA knockdown cells. The intercellular number of *C*. *jejuni* cells had increased, as compared with cells transfected with the sh-control vector (Fig 3). In addition, the addition of pharmacological inducers of ER stress and *C*. *jejuni* infection induced UPR-attenuated *C*. *jejuni* invasion (Figs 4 and 5). Also, the suppression effect of UPR signaling in intracellular invasion was not found in S. Enteritidis infection or FITC-dextran intake (Fig 6A). Taken together, ER stress and the accompanying increase in UPR signaling appear to be defense responses against *C*. *jejuni* invasion (Fig 7).

The focus of this study was the effect of UPR signaling on *C*. *jejuni* infection; thus, the infection period was determined according to the maximal bacterial invasion (data not shown). The data showed that *C*. *jejuni* infection induced CHOP production and phosphorylation of eIF2α, but splicing of XBP1 and cleavage of ATF6 were not observed. While UPR signaling is controlled by 3 ER-transmembrane proteins (PERK, IRE1, and ATF6), the induction of the PERK pathway in *C*. *jejuni*-infected cells was monitored (Fig 2). These results raise the possibility that the PERK-eIF2α signaling pathway was selectively activated, and CHOP expression was up-regulated by *C*. *jejuni* infection for 12 h. However, long-term infection of more than 12 h may activate another UPR pathway, which induces a stronger effective defense response against *C*. *jejuni* infection. Furthermore, it is known that persistent ER stress leads to inflammation-associated apoptotic cell death, which has an adverse effect in the host. Thus, long-term infection studies could reveal the mechanisms of inflammation and apoptosis induction during *C*. *jejuni* infection.

It has been revealed that several bacterial species activate UPR signaling in host cells upon infection. The major group of bacterial toxins capable of inducing UPR includes the AB_5_ toxins, such as Shiga toxin [43], subtilase cytotoxin of Shiga-toxigenic *Escherichia coli* [44], and cholera toxin. Infection of *Vibrio cholerae* [45] directly induces ER stress by interacting with ER chaperone or sensor proteins, which can induce UPR by short-term treatment with toxins (fewer than 6 h). Such severe stress damages or disrupts homeostasis of host epithelial cells, which leads to bacterial infection. During long-term (12 h) *C*. *jejuni* infection, host cells are gradually damaged and UPR signaling is activated. But according to genomic studies, the genome of *C*. *jejuni* does not code for a homologous AB_5_ toxin and *C*. *jejuni* did not induce severe injury to the host epithelial cells.

On the other hand, UPR is induced by secretion of bacterial toxins, such as the pore-forming toxins, including aerolysin of *Aeromonas hydrophila*, Cry5B of *Bacillus thuringiensis* [46], listeriolysin O of *Listeria monocytogenes* [25], and the small-protein early secretory antigenic target 6 of *Mycobacterium tuberculosis* [28]. These toxins can activate UPR signaling through perturbation of Ca^2+^ homeostasis, activation of mitogen-activated protein kinase, or production of reactive oxygen species. Each of these signal transduction pathways were shown to decrease bacterial survival in host cells. Interestingly, group A streptococcus (GAS) induces UPR to capture asparagine (ASN) from the host cells and enhance its rate of proliferation [47]. PERK-eIF2α pathway is induced by GAS releasing hemolysin toxins, streptolysin O and streptolysin S, and upregulates the transcription of ASN synthetase. It was reported that *C*. *jejuni* have the mechanism to utilize ASN produced by host cells for survival strategy [48]. PERK-eIF2α pathway was also activated by *C*. *jejuni* infection, but the survival rate of intracellular *C*. *jejuni* was not changing during ER stress (Fig 5F). In this study, we did not check the effect of ER stress on extracellular *C*. *jejuni*. Taking these reports into account, it is possible that ER stress is activated for the extracellular *C*. *jejuni*.

In the present study, the activation of UPR pathway decreased bacterial invasion. This inhibition of bacterial invasion was associated with the lipid raft-mediated endocytic pathway. A recent report indicated that the PERK signaling pathway modulated ER-plasma membrane (PM) contact sites by inducing changes to the actin skeleton [49]. Modulation of the ER-PM contact sites allows the ER to sense and respond to changes in the PM components, such as the quantity and types of lipids and proteins, which leads to the induction of several signaling pathways [50]. Hence, it can be considered that these ER-PM contact sites influence the *C*. *jejuni* invasion processes, endocytosis-like uptake, and microtubule-dependent cellular transport.

In this study, it was not possible to identify the factors responsible for the activation of UPR in *C*. *jejuni;* thus, the underlying mechanisms remain unclear. Nonetheless, UPR was induced by the invasion-deficient *C*. *jejuni* mutant strain as well as the WT strain. It is likely that other virulence factors, such as cytolethal distending toxins (CDT) or lipooligosaccharides, are related to UPR induction during *C*. *jejuni* infection [51, 52]. In quite recent international Campylobacter, Helicobacter and Related Organisms conference, it was reported that CDT activated IRE1-XBP1 pathway of the UPR [53]. In this study, however, we could not detect the activation of IRE1-XBP1 pathway in *C*. *jejuni* infected cells, because of the difference of experiment model. And our results suggest the presence of another ER stress activating factor, and it is involved in activation of PERK-eIF2α pathway in *C*. *jejuni* infection. Further studies are obviously needed to reveal the contribution of virulence factors in *C*. *jejuni*-induced ER stress. In addition, phosphorylation of eIF2α is known as an integrated stress response-signaling factor [54]. It has been reported that eIF2α is phosphorylated by not only PERK activation during ER stress, but also other stress-response signaling molecules, such as general control nonderepressible 2, under conditions of amino acid starvation [55, 56], heme-regulated eIF2α kinase during iron deficiency [57], and protein kinase RNA during viral infection [58–60]. To confirm the contribution of ER stress in eIF2α-phosphorylation, it is necessary to investigate the role of PERK-eIF2α during *C*. *jejuni* infection.

We found that ER stress is induced during *C*. *jejuni* infection and UPR system a defense strategy of host cells against *C*. *jejuni* infection. Our data suggests that UPR affects the early step of bacterial infection. Those results raise the possibility that ER stress could be an effective therapeutic target for the treatment of *C*. *jejuni* infection.

## Supporting information

S1 Fig The UPR was activated *by C*. *jejuni* infection in various types of cells. (A) Western blotting of UPR signaling proteins in Caco-2 cells infected woth *C*. *jejuni* 81–168 strain was conducted. (B and C) It was also conducted in HeLa cells or in HT-29 cells. Each experiment was repeated three times.
(TIF)

S2 Fig UPR signaling did not affects adhesion or intracellular bacterial survival. (A and B) The bacterial adhesion and intracellular survival in HeLa cells transfected with the pENTR-PERK vector, the pENTR-IRE1 vector, the pENTR-ATF6 vector, or a non-target shRNA control vector were assessed after 1 or 3 h of *C*. *jejuni* infection using the gentamycin protection assay. Each experiment was repeated three times.
(TIF)

S3 Fig The suppression effect of ER stress on intracellular intake is specific to *C*. *jejuni*. Caco-2 cells were pretreated with thapsigargin (2.5 μM), tunicamycin (5.0 μg/mL), or DMSO for 12 h and infected with *C*. *jejuni* for 6 h, with *S*. Enteritidis for 1 h, or treated with 0.1 mg/mL 10 kDa FITC-dextran for 2 h. Each intracellular number were evaluated as in (Fig 6A). Each experiment was repeated three times.
(TIF)
