# Supplementary figures and images for: Host cellular unfolded protein response signaling regulates Campylobacter jejuni invasion
Source: PLoS One. 2018 Oct 15;13(10):e0205865. doi: 10.1371/journal.pone.0205865 (PMC6188877; doi:10.1371/journal.pone.0205865)

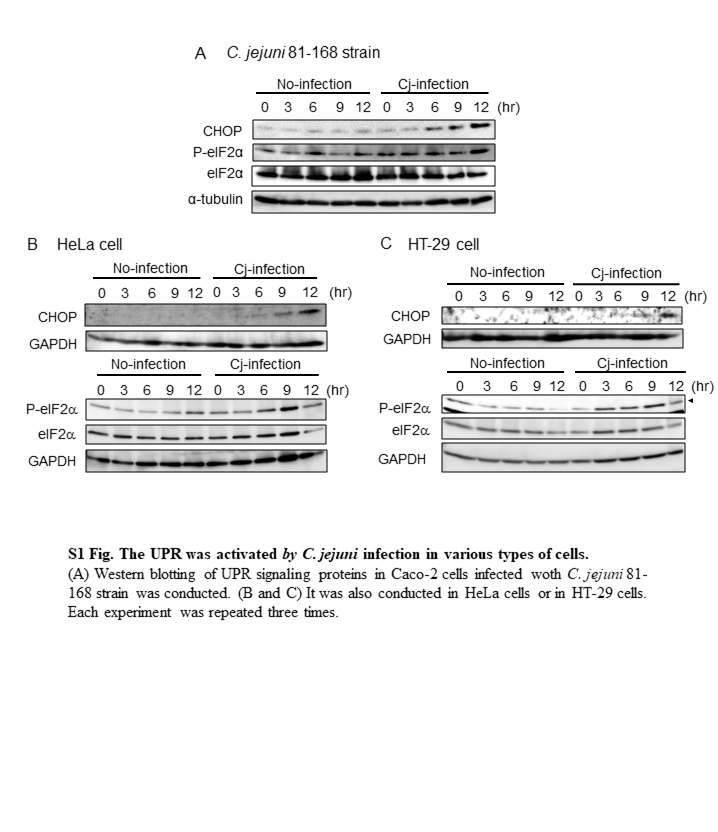

Supplement: S1 Fig — (A) Western blotting of UPR signaling proteins in Caco-2 cells infected woth C. jejuni 81–168 strain was conducted. (B and C) It was also conducted in HeLa cells or in HT-29 cells. Each experiment was repeated three times. (TIF) [file pone.0205865.s001.TIF]

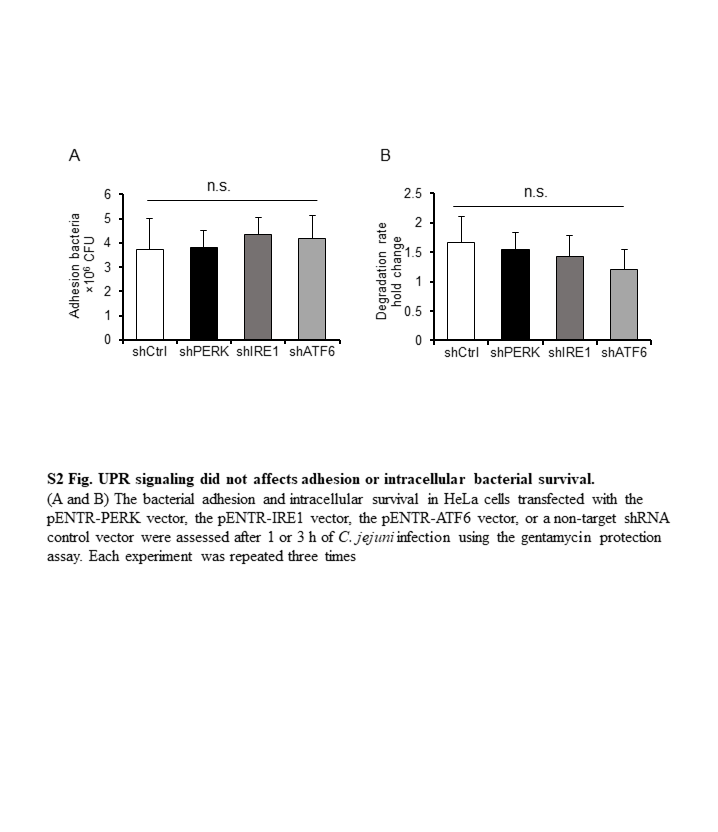

Supplement: S2 Fig — (A and B) The bacterial adhesion and intracellular survival in HeLa cells transfected with the pENTR-PERK vector, the pENTR-IRE1 vector, the pENTR-ATF6 vector, or a non-target shRNA control vector were assessed after 1 or 3 h of C. jejuni infection using the gentamycin protection assay. Each experiment was repeated three times. (TIF) [file pone.0205865.s002.TIF]

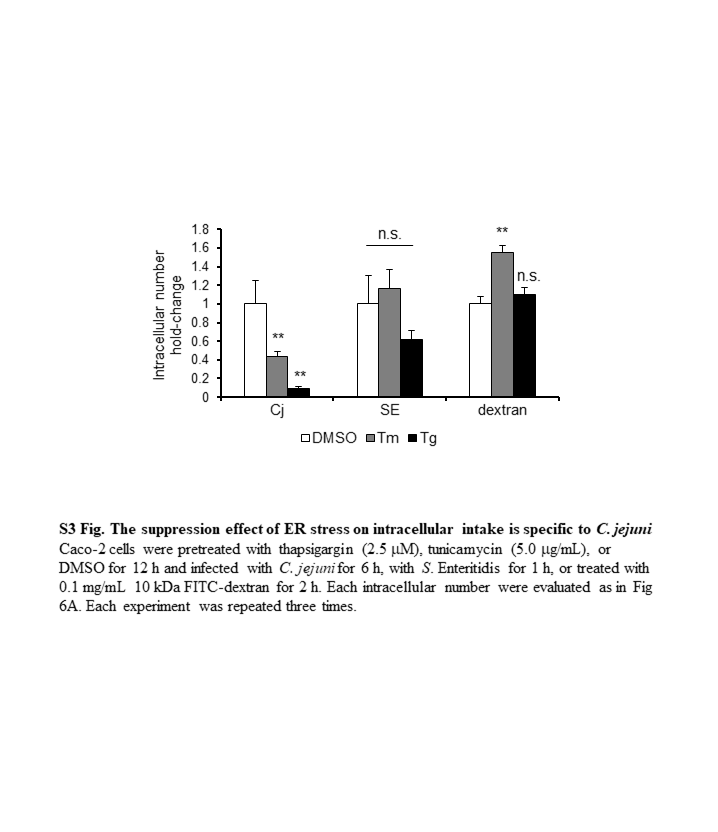

Supplement: S3 Fig — Caco-2 cells were pretreated with thapsigargin (2.5 μM), tunicamycin (5.0 μg/mL), or DMSO for 12 h and infected with C. jejuni for 6 h, with S. Enteritidis for 1 h, or treated with 0.1 mg/mL 10 kDa FITC-dextran for 2 h. Each intracellular number were evaluated as in (Fig 6A). Each experiment was repeated three times. (TIF) [file pone.0205865.s003.TIF]
